# Plant behaviour in response to the environment: information processing in the solid state

**DOI:** 10.1098/rstb.2018.0370

**Published:** 2019-04-22

**Authors:** Salva Duran-Nebreda, George W. Bassel

**Affiliations:** School of Biosciences, University of Birmingham, Birmingham B15 2TT, UK

**Keywords:** information processing, computational networks, plant, connectome, biological computation

## Abstract

Information processing and storage underpins many biological processes of vital importance to organism survival. Like animals, plants also acquire, store and process environmental information relevant to their fitness, and this is particularly evident in their decision-making. The control of plant organ growth and timing of their developmental transitions are carefully orchestrated by the collective action of many connected computing agents, the cells, in what could be addressed as distributed computation. Here, we discuss some examples of biological information processing in plants, with special interest in the connection to formal computational models drawn from theoretical frameworks. Research into biological processes with a computational perspective may yield new insights and provide a general framework for information processing across different substrates.

This article is part of the theme issue ‘Liquid brains, solid brains: How distributed cognitive architectures process information’.

## 1. Introduction

### (a) Information processing in biological systems

Biological entities face complex and challenging environments, where successfully making use of past experiences or being able to make accurate predictions about the future can make a difference in their survival and reproduction.

Drawing from man-made archetypes of information processing, organisms are said to contain a non-explicit model of the environment they exist in [1]. This provides organisms with a mechanism to transform sensed environmental variables into a usable prediction that informs decision-making. Thus, biological systems compute: they acquire, store, process and act on information that surrounds them [2,3]. This is obviously not exclusive to higher order organisms but observed at multiple scales: from homeostasis maintenance at the physiological level to developmental transitions at the cellular level [4,5] and complex goal-oriented decision-making at the organism level [6,7].

There are several key differences with man-made computational devices that prevent us from fully mapping analogies between biological and machine computation. Both are embedded in a physical reality but only cells face the constraints imposed by homeostasis (the tendency towards a fixed point equilibrium of system components, maintained by physiological processes) and the ability to replicate oneself (autopoiesis). This requires an extra set of ‘processes’ to be executed in the background, which can interfere with or override other information processes.

Secondly, in striking difference from digital electric pulses running through logic gates, biological information is encoded in many different forms, which need to be translated between formats [8,9]. The range of information transducing formats goes from proteins, ions or mRNAs moving from cell to cell, to organism-wide signals like mechanical stresses and hormones that organize major developmental events. These transitions in the nature of communicated information create boundaries as to how and where information can be appropriately interpreted. Additionally, this means that information travels at different speeds depending on the substrate and mechanisms for transport, from passive diffusion to the rapid ion channel de-polarization.

Plants are no exception to the constraints and characteristics that we have just introduced [10]. Plants are made of immobilized cells, creating as solid-state substrate in which information flows from cell to cell. They are subjected to highly fluctuating environments, and must process complex environmental cues in order to optimize the timing of the key transitions of their life cycle. In this manuscript, we will review a series of examples of biological processes found in plants, analysed from the perspective of information processing, highlighting the benefits and pitfalls of taking such a point of view.

### (b) Biological information processing in non-neuronal systems

Similar to computation in engineered systems, information processing in biological systems requires storage, transfer and processing of information so that it can be converted into a usable form [2]. The most used example for these processes is the animal nervous system, which can harbour up to billions of dedicated cells to perform such tasks. However, these processes not only happen in neurons, but at diverging scales through organisms. In this section, we include some examples of information processing at very different biological scales; these provide the underlying ideas and methods to better understand information processing in plants.

At the cellular scale, gene regulatory networks (GRNs) can also compute [11]. The dynamical system composed of protein concentrations, DNA sequences and their interactions can simulate computations best described as memory bounded Turing machines [12]. These are equivalent to a limited version of Turing's proposed scheme of universal computation [13] and are capable of simulating any other computing device.

Other clues into the universality of biological computing designs come from research into biological motifs [14,15]. Taking a network perspective of organization of regulatory elements, collections of devices can be classified by architectural or behavioural properties. This provides us with a general framework with which we can compare dynamical behaviours without the minutiae of the particular substrate used to implement it. Interestingly, when freed from such constraints, we can compare families of devices and provide a solid rationale for the designs observed in nature [15]. The driving pressure behind particular motifs can be robustness to signal noise, evolvability or simplicity in the number of operating components. We can use these universal constraints to better understand what can be expected from evolved architectures as well as inform our own engineering endeavours.

**Figure 1.**
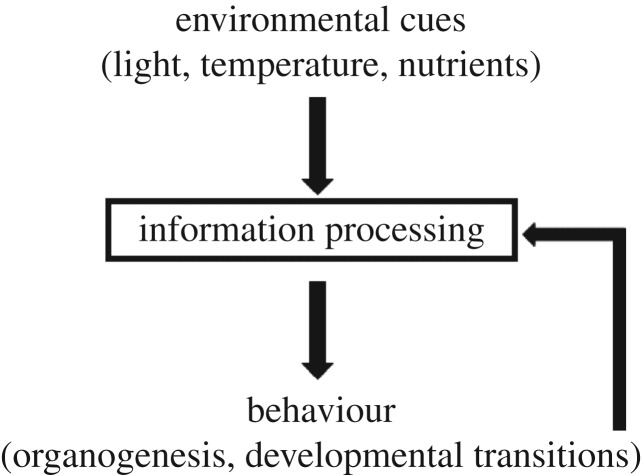
Information processing loop in multicellular plants. Environmental cues are integrated and prompt changes in plant behaviour in the form of developmental transitions and organogenesis. The creation of new organs, the substrates of computation in plants, in turn feed back onto system-level information processing. An indeterminate loop forms by information processing, leading to the creation of new organs, which in turn process information themselves.

Developmental processes can also be regarded as information processing [16,17]. They require gathering information about both the internal state of the organism and external physical cues that can trigger these developmental events, and they produce an output that is a transformation of the computational substrate through a feedback loop (figure 1). This means that the ‘hardware’ is not constant; instead, new physical computing units are produced as a result of information processing. The new biological form might be able to perform new operations and has its internal state changed, so it perceives different information. The perspective of development as a computational scheme has been explored extensively [16–21], with direct translation to real computational problems that are not trivial to solve. A recent perspective on the causal loop comprising genetic programmes, developmental induction and tissue geometry can be found in [21], which challenges the view of organisms or the physical embodiment of biological entities as a mere epiphenomenon of genetic programmes.

Another well-studied class of organism that performs non-neuronal information processing is the slime mould *Physarum polycephalum.* Experiments have been performed using this creature to investigate its ability to execute computational tasks, such as navigating a maze, and finding the shortest path between food sources [22]. The slime mould will often find a ‘good-enough’ solution to this problem, and not necessarily that which is optimal. The output is sufficient to support the growth of the individual in the absence of additional selective forces. This illustrates an important aspect of computational outputs in biology, which may not always be optimal.

### (c) Centralized versus distributed computation

Artificial computation in our personal computers (PCs) relies, for the most part, in a central processing unit (CPU)-based architecture. This means that there is a main computational device through which information flows and where global processes are run. This generates the so-called bus bottleneck: a limiting factor for computation can be the bandwidth or amount of information that can be fed into the CPU. A different architectural organization was proposed to overcome the bus bottleneck and CPU design constraints: distributed computing [23,24]. In this form of information processing, several processing units (PUs) exist, acting in parallel and performing lower-level tasks that are allocated to them instead of global functions. Then, the results of lower-level calculations have to be aggregated, leading to a solution to the global problem from these smaller problems. This ensures that some bottlenecks and CPU complexity constraints can be avoided but also generates new challenges to be faced: allocation and coordination of the PUs become core issues to be resolved.

There are other advantages to using a distributed architecture. Operating with redundant components means the system is more resilient to the random failure of parts [25–27], as no single PU is responsible nor necessary for the operation of the system as a whole. Computational power can also be increased without the need to increase the complexity of the PUs [28,29]. Adding new computational elements or rearranging the connections among them enables new tasks to be performed, providing adaptability and evolvability [23].

Distributed architectures are found in many complex biological systems, from bacterial colonies [30–32] to social insects and other animal groups [33–35]. In order to understand a distributed system operation, special focus needs to be placed on the mechanism(s) employed to aggregate, coordinate action and communicate internal states. This includes sensory apparatus in animals [31], pheromone trails in social insects [35] and diffusible chemicals for bacterial colonies [32]. It has been shown that isolated systems can be pushed into collective dynamics by tampering with their communicating and sensory mechanisms [36,37]. In turn, better understanding of behaviour and operation of natural distributed computing systems has inspired the development of faster, more robust algorithms for computer science [34], providing also the basis for deep insights and a more fundamental theory of computational systems.

**Figure 2.**
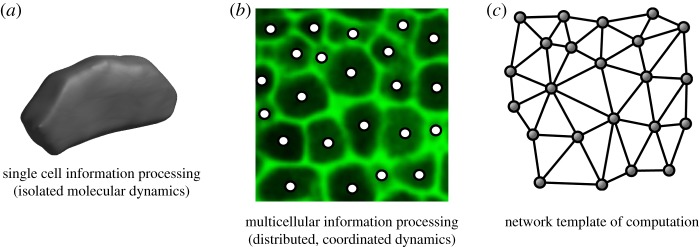
(*a*) Single cells are able to perform computations using their intracellular dynamics. These are integrated at the higher scale in the context of a tissue (*b*) by means of aggregation. The aggregation process makes use of the network structural template in which transport takes place. (*c*) Network structural template depiction of the tissue in (*b*). (Online version in colour.)

Organs in general, including those from plants, operate under similar conditions to distributed computing. They do not rely on a single CPU/cell to govern action, instead goal-oriented behaviour is broken down into smaller tasks and allocated into the many constituent cells [38] (figure 2*a,b*). A consensus such as determining organ size or timing for a developmental transition is then reconstructed from the small-scale cellular contributions by aggregation. This happens in parallel, with many processes and cells contributing to the final outcome of the computation. It is at this level that we often lack knowledge of how thousands of asynchronous cellular internal dynamics coalesce into collective decision-making that pervades organ function. With multiple cells coming together and connecting to form a computing tissue, topology and organization of major information routes provide an additional set of constraints to consider. This creates the major differences between a GRN operating in a single cell and several GRNs embedded in a complex tissue.

A useful perspective to analyse the distributed and self-organized nature of organ behaviour is Smith's [39] view of developmental processes. Similar to the CPU versus distributed architecture dichotomy, developmental processes can be classified into two opposing types, stamp-like and self-organized mechanisms, depending on the nature of the flow of information within a system. Stamp-like mechanisms rely on hierarchical relations among the lower-level components, with some cells organizing and providing information for the rest of the tissue to follow (i.e. gradient-based positional information specification of development [40]). More closely related to distributed architectures, self-organized developmental processes rely on collective action by a set of similar agents, with horizontal information transfer among the computing agents. A complementary perspective of developmental processes can also be found in [41].

The horizontal nature of distributed architectures poses additional challenges to the design and operation of organs; namely, the additional costs of coordinating and communicating results to other cells through diffusible molecules [42]. This is especially true of the cellular arrangement in plant tissues, which is lattice-like. Owing to the mechanical interactions between adjacent cells and their immobility relative to one another, the diversity of cell shapes is limited, leading to this constraint in the way in which the cells are arranged. As a consequence, there are a limited number of shortcuts for information transfer, and molecules need to traverse a vast array of cells to reach their potential destination [43] (see figure 2*b* for an example of a lattice-like tissue). This constraint is reduced in heterogeneous cellular networks of some plant organs where enlarged cells connect physically vastly separated regions and provide a backbone for centralized information flow, bringing together cells and tissues that can be far apart. Thus, topological arrangement of the computing elements as well as the establishment of shortcuts that enable fast transfer of information become important constraints in organ design and function. Below we discuss several examples of centralized and distributed computation in plant organs, with special interest in their relation to standard computational models and theory.

## 2. Discussion

### (a) Examples of distributed computation in plants

#### (i) Distributed control of gas exchange in leaves through stomata

Leaves regulate gas exchange across their surfaces by opening and closing pairs of guard cells and pore (stoma) aperture [44]. When stomata are open, gas exchange can occur. This however comes at the expense of accelerated water loss from the tissue. Opening stomata imposes an important trade-off between gas and water relations that needs to be successfully manage in order to optimize plant fitness.

**Figure 3.**
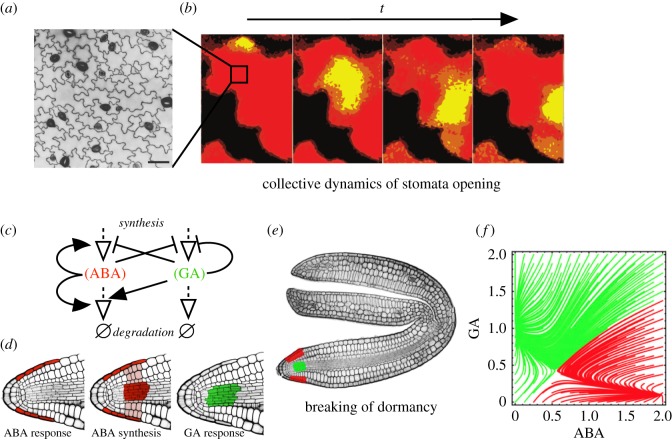
Multicellular information processing in plants. (*a*) Stomata (dark cells) are dynamically open and closed in order to capture CO_2_ and avoid excessive loss of water. (*b*) Thermal images of leaf surfaces showing the current state of stomata aperture within sectors. Patches of coordinated stomata activity are seen, indicating that collective dynamics of stomata are present in the form of excitable media-like waves that propagate through the leaf surface. (*c*) The hormone metabolic network underpinning the regulation of abscisic acid (ABA) and gibberellic acid (GA) levels in dormant *Arabidopsis* seeds. (*d*) Distribution of ABA and GA synthesis and response within distinct cell types of the dormant embryo radicle. (*e*) Spatial sites of ABA and GA responses within the dormant *Arabidopsis* embryo. (*f*) Attractor dynamics of the hormone metabolic interaction network in dormant *Arabidopsis* seeds when the distinct spatial embedding of hormone responses is taken into account. (*a,b*) taken from [45] and (*f*) from [46].

It has been shown that the stomata of cocklebur (*Xanthium strumarium*) do not operate separately, but in patches of the same state (open or closed) [45]. It was suggested that local interactions among stomata could propagate in a coordinated fashion and create a higher order phenomenon similar to travelling waves of stomatal state across a leaf surface [45] (figure 3*a,b*). The collective dynamics of stomata in leaf surfaces were likened to cellular automaton (CA) theory. CAs are space-embedded models with collections of interconnected cells, each with a discrete internal state [47]. Using the CA's rules, the internal state is dynamically updated, using only the current cellular state and that of the cell's immediate neighbours [47].

Peak and coauthors showed that a CA density Gács–Kurdyumov–Levin classifier [45,48] can be used to model the establishment and spatial propagation of same-state domains in leaf surfaces. This proposes that stomata can sense the open–closed state of their neighbours and switch to match theirs, and is statistically similar to the random accumulation of grains of sand in a lattice [49]: gas accumulation provides the instability for stomata to open, driving the critical propagation of open domains through the leaf surface. Such avalanches of activation can be modelled as a product of a self-organized critical system [49,50], and show some characteristic distributions for avalanche sizes and waiting times between events closely matching the experimental results. Self-organized criticality is typically found in driven systems, which experience a constant input of energy, and from a dynamic systems perspective have a critical point as an attractor [49].

#### (ii) Fluctuating temperatures are integrated with distributed computation and control seed dormancy

Plant seeds remain dormant awaiting the opportune conditions for germination [51]. The GRNs underpinning this process are regulated by the two antagonistically acting hormones: abscisic acid (ABA) and gibberellic acid (GA), promoting dormancy and germination, respectively [51,52] (figure 3*c*). A ratio threshold of hormone abundance is used to take the irreversible decision to commence germination. It has been recently shown that the response to these molecules is spatially segregated within a dormant *Arabidopsis* embryo [46]. Both centres control the metabolism of ABA, but response feedbacks remain separated in distinct cell types (figure 3*d–f*).

This control logic of two separated control centres that mutually inhibit each other by mobile agents is also found in the human brain. The ganglia–cerebellum–cortex loop [53,54] is involved in the control of motor centres, playing an important part in movement decision-making. In human brains, this motif of mutually inhibiting centres is thought to filter out noise by introducing a time delay. In *Arabidopsis*, this spatial separation was shown to perform an opposite function in harnessing variable inputs [46], promoting germination when the daily temperature fluctuations increase [55].

As such, the system displays alternate states that can be viewed as attractors on a larger variable space (figure 3*f*). Attractor dynamics and ODE formulations can be used to better understand the behaviour of the system as well as offer interesting opportunities for intervention and engineering [46].

This example provides an interesting perspective on conserved computation motifs [15,16] that might be found across domains and species. From computers to synthetic biology to real biology [16], similar designs can be found that are either very robust to signal noise or highly evolvable. Such pervasiveness suggests that interdisciplinary research might be able to translate knowledge obtained from other substrates, as well as design principles and limitations.

A further parallel in the control of this system with distributed computation is observed by altering the rate at which the distinct hormone response centres communicate in a dormant seed. Increasing the expression of the *NFP3* transporter, which moves both ABA and GA, increases the sensitivity of seeds to alternating temperatures in the breaking of seed dormancy [46]. The altering of this aggregation rate therefore impacts the outputs of the system, as observed in computational systems.

#### (iii) Distributed control of flowering in response to cold temperatures

Similar to seed germination control, the timing of the induction of flowering is a crucial decision in the plant life cycle [56]. A plant needs to make a prediction on when is the optimal timing to create reproductive organs; this includes integrating environmental information like future resource availability, as well as flowering when other plants of the same species undergo this transition.

In many species, flowering is initiated by exposure to cold temperatures in a process called vernalization. In the case of *Arabidopsis*, the repressor gene *FLOWERING LOCUS C* (*FLC*) mediates this response through epigenetic silencing [57,58]. The average expression of *FLC* follows a very predictable continuous decline in response to cold. Upon closer inspection, individual cells were found to be either silenced or non-silenced, leading to the proposal that cold is being digitally registered in individual cells of the plant [59].

Collectively, the individual binary state of each cell needs to be aggregated in a global response, providing a robust estimation of environmental temperature. The molecular mechanisms by which this aggregation takes place are still unknown, although some mobile genetic elements controlling flowering have been described, such as *FLOWERING LOCUS T* [60]. At the theoretical level, we can assume that this mechanism probably involves a message passing algorithm, where each cell tries to communicate its state to the neighbouring ones and a majority-rule is applied to decide when a critical fraction of cells have transitioned [61].

Recent work has demonstrated the *FLC-*based cold registration silencing mechanism to take into account alternating temperatures in the control of flowering time [62], as demonstrated in the control of seed dormancy. This suggests this complex transformation of temperature inputs is conserved across diverse transitions in plant development.

#### (iv) Dynamic topological rearrangement of shoot apical meristem domains affects distributed computation

**Figure 4.**
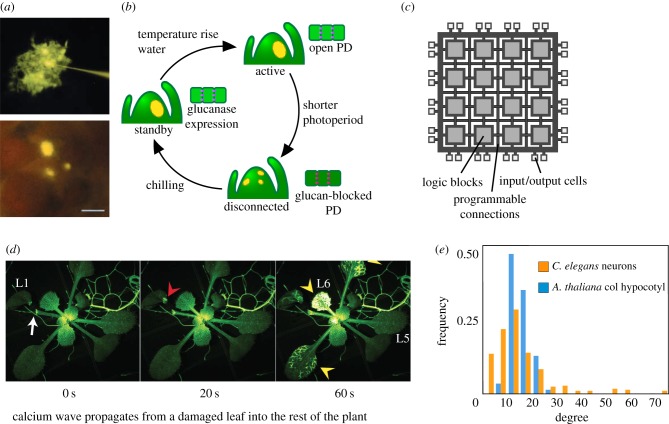
Multicellular information processing in plants. (*a*) In birch, SAMs can be formed of a collection of isolated cells, or a fully connected and communicating tissue, depending on environmental cues. (*b*) A fragmented system does not promote the growth activation of the meristem, thus following a different decision-making scheme from a fully connected SAM. (*c*) FPGA design with multiple PUs that can be dynamically rearranged in order to manipulate the computation. (*d*) Glutamate mediates calcium waves that rapidly propagate in *Arabidopsis thaliana*. These waves make use of topological shortcuts in organ design. (*e*) Degree distributions in *Caenorhabditis elegans* neurons and *A. thaliana* hypocotyl cells. Although showing similar average values, *C. elegans* neurons show a long-tailed distribution compared with the lattice-like *Arabidopsis* hypocotyl. Greater cell degree is possible owing to the intricate shapes that neuronal animal cells can attain. (*a*) taken from [63], and (*d*) taken from [67]. (Online version in colour.)

In a seminal paper in birch (*Betula pubescence*) [63], it was shown that the shoot apical meristem (SAM) of this species undergoes a ‘fragmentation’ process that is dependent on seasonal biological activity. During the winter, when apical growth is arrested, cells in the SAM are isolated in terms of their communication with other parts of this plant. This is achieved by modifying the aperture of cytoplasmic channels located at the interface between cells called plasmodesmata (PD) [64]. Doing this in a coordinated manner leads to the creation of small connected collections of cells, termed symplastic domains [65,66]. The presence of symplastic domains in the birch SAM during the winter was shown by injecting a freely moving dye in different locations and tracking its movement (figure 4*a*). By contrast, the cells in the SAM during active growth in the summer are communicating readily, with the dye propagating from any starting point to any other cell given enough time.

An interesting computational architecture that shares some properties with the system described by Rinne & van der Schoot [63] is that of field programmable gate arrays (FPGAs). FPGAs are electronic circuits capable of changing the computation being carried out by altering the connections between logic blocks inside of them (see figure 4*c* for a rendition of an FPGA) [68]. This allows FPGAs to be adaptable and to implement newly developed algorithms. In the case of the birch SAM, the dynamic rearrangement of the symplastic domains provides the same multicellular template with distinct connectivity states, each running separate developmental programmes: a resting state where only autopoietic functions are implemented and an active state where growth and new organs are created. This is supported by recent work in hybrid aspen which showed season-dependent cellular connectivity in the SAM of this species to regulate the release from bud dormancy [67].

Rinne & van der Schoot [63] suggested the symplastic domains might provide cues to the establishment of new above-ground organs, dynamically setting the boundary conditions of the template, as well as controlling the size of the computing agent population (figure 4*b*). The critical contribution of size and/or density thresholds to behavioural changes has been thoroughly explored in other distributed systems, from bacteria [69] to animal communities [33,34], and may also be acting in this context.

### (b) Speed versus robustness in multicellular information processing

Plant tissues are solid masses made of rigid, space-filling cells [43]. This severely constrains the design space in terms of network topologies that is available to tissue formation [38]. Isotropy and lattice-like organs (figure 2*b*) are frequently found in plants; this constitutes a limiting factor in how fast information can be transmitted through plant organs [70].

Network theory can help us better understand distances in plant tissues by considering average walks taken to travel between two random nodes [70]. At the other end of the spectrum, tissue architectures with elongated cells that connect with many other cells give rise to heterogeneous networks, which are known to reduce average distances between two random nodes in the network. This is achieved at the cost of robustness; when random or targeted failure of components is applied to heterogeneous networks they break down more quickly than lattice-like ones [71]. Heterogeneous designs include vascular systems, which create shortcuts in the network that facilitate long-distance transport of molecules.

This intrinsic trade-off between speed of information transfer and robustness to random noise provides us with a coherent framework from which we can understand optimization in tissue architecture. Plant organs do not need to adhere to a single principle of design: some parts of the plant might include lattices while others show clear signs of long-range transportation optimization.

### (c) Long-range rapid information transfer in plants

It has been recently shown [72–74] that plants also possess a rapid information distribution mechanism that uses small molecules and a vast network of highly connected cells to communicate cellular state. In an example experimental condition, this communication was triggered when one leaf of the plant was damaged by a caterpillar, and a signal was transmitted through the vascular system [72–74] (figure 4*d*). The reported rate of transmission was roughly 1 mm s^−1^, orders of magnitude slower than the speed of information transfer in animal nervous systems.

These findings provide a mechanism for the central integration of information across a plant. But what are the computational consequences of such a design? A global network of information transfer allows synchronization of states in the whole organism. In a matter of seconds, collective responses can be orchestrated to environmental challenges, and coupling in temporal dynamics becomes easily attainable. In terms of fitness impacts, this means that the whole plant can adequately respond to the caterpillar aggression and better its chances of survival. This mechanism of burst information transfer in plants challenges the common view of plants as temporally slow and computationally basic organisms.

### (d) Connectionist approaches to understanding multicellular information processing in plants

Topology and communication are the major constraints in the operation of distributed systems. Accordingly, fully characterizing the template in which information processing takes place can provide us with defining information on organ behaviour. The presence of shortcuts for information transfer and the global connectivity of the system are signatures of information processing events inside tissues. Structural cellular interaction mapping is especially relevant in plants because plant cells are immobilized and limited in the shapes they can attain compared with animal organs, especially neuronal systems (figure 4*e*), where interactions are dynamic. PD, however, slightly alter this picture. Even though the template cannot be changed through cell migration, the ‘channel bandwidth’ of communication between cells can be tuned, providing extra functionality and adding a dynamic component to an otherwise static design, following the principle of an FPGA.

This structural mapping should be complemented when possible with functional information about the network [71]. This means both node (cells) and edge (interfaces) annotation. With cell types and relevant molecule concentrations included in the model, we can better understand how an organ was constructed and generated through development, as well as the inherent complexity present in the tissue. Annotation allows us to bridge scales between molecular networks and cellular networks by stimulating growth dynamics and multiscale phenomena, providing us with extended predictive capabilities in terms of dynamics and thus computation.

## 3. Conclusion

In this paper, we have discussed how collection of cells process information in plants, with special interest in distributed computation and their connection to computing theory. Taking such a perspective provides us with a framework to close a knowledge gap between the lower-level molecular interactions and higher order adaptive behaviours. Additionally, it has been shown that research into naturally occurring information processing drives the creation of faster more robust algorithms [34,75,76] useful for the computer sciences. Also, understanding information processing on natural substrates can provide computer scientists with new insights into efficient, constrained computational systems. Finally, comparing how information processing works in non-neuronal systems may also provide cross-fertilization for brain connectionists, helping build a general theory of multicellular information processing in diverse biological systems.

## Data accessibility

This article has no additional data.

## References

[1] Holland JH 1995 *Hidden order: how adaptation builds complexity*. Cambridge, MA: Perseus.

[2] Lyon P 2015 The cognitive cell: bacterial behavior reconsidered. Front. Microbiol. 6, 264 (10.3389/fmicb.2015.00264) 25926819 PMC4396460

[3] Mitchell M 2011 Ubiquity symposium: biological computation. Ubiquity 2011, 3 (10.1145/1922681.1925843)

[4] Wiener N 1961 Cybernetics or control and communication in the animal and the machine, vol. 25 Cambridge, MA: MIT Press.

[5] Johnson EO, Kamilaris TC, Chrousos GP, Gold PW 1992 Mechanisms of stress: a dynamic overview of hormonal and behavioral homeostasis. Neurosci. Biobehav. Rev. 16, 115–130. (10.1016/S0149-7634(05)80175-7) 1630726

[6] Simões LF, Cruz C, Ribeiro RA, Correia L, Seidl T, Ampatzis C, Dario Izzo D 2011 Path planning strategies inspired by swarm behaviour of plant root apexes. Ariadna Final Report. Report no. 09/6401. European Space Agency.

[7] Hutchings MJ, Slade AJ 1988 Morphological plasticity, foraging and integration in clonal perennial herbs. Symp. Ecol. Soc. **1988**.

[8] Laughlin SB, van Steveninck RRDR, Anderson JC 1998 The metabolic cost of neural information. Nat. Neurosci. 1, 36 (10.1038/236) 10195106

[9] Tu Y 2013 Quantitative modeling of bacterial chemotaxis: signal amplification and accurate adaptation. Annu. Rev. Biophys. 42, 337–359. (10.1146/annurev-biophys-083012-130358) 23451887 PMC3737589

[10] Trewavas A 2003 Aspects of plant intelligence. Ann. Bot. 92, 1–20. (10.1093/aob/mcg101) 12740212 PMC4243628

[11] Kauffman SA 1969 Metabolic stability and epigenesis in randomly constructed genetic nets. J. Theor. Biol. 22, 437–467. (10.1016/0022-5193(69)90015-0) 5803332

[12] Ben-Hur A, Siegelmann HT 2004 Computation in gene networks. Chaos 14, 145–151. (10.1063/1.1633371) 15003055

[13] Turing AM 1937 On computable numbers, with an application to the Entscheidungs problem. Proc. Lond. Math. Soc. 2, 230–265. (10.1112/plms/s2-42.1.230)

[14] Milo R, Shen-Orr S, Itzkovitz S, Kashtan N, Chklovskii D, Alon U 2002 Network motifs: simple building blocks of complex networks. Science 298, 824–827. (10.1126/science.298.5594.824) 12399590

[15] Schaerli Y, Munteanu A, Gili M, Cotterell J, Sharpe J, Isalan M 2014 A unified design space of synthetic stripe-forming networks. Nat. Commun. 5, 4905 (10.1038/ncomms5905) 25247316 PMC4172969

[16] Alberch P 1989 The logic of monsters: evidence for internal constraint in development and evolution. Geobios 22, 21–57. (10.1016/S0016-6995(89)80006-3)

[17] Baluška F, Levin M 2016 On having no head: cognition throughout biological systems. Front. Psychol. 7, 902 (10.3389/fpsyg.2016.00902) 27445884 PMC4914563

[18] Adamatzky A, de Lacy Costello B, Shirakawa T. 2008 Universal computation with limited resources: Belousov–Zhabotinsky and Physarum computers. Int. J. Bifurcation Chaos 18, 2373–2389. (10.1142/S0218127408021750)

[19] Leyser O 2009 The control of shoot branching: an example of plant information processing. Plant Cell Environ. 32, 694–803. (10.1111/j.1365-3040.2009.01930.x) 19143993

[20] Prusinkiewicz P, Lindenmayer A 2012 The algorithmic beauty of plants. Berlin, Germany: Springer Science & Business Media.

[21] Ollé-Vila A, Duran-Nebreda S, Conde-Pueyo N, Montañez R, Solé R 2016 A morphospace for synthetic organs and organoids: the possible and the actual. Integr. Biol. 8, 485–503. (10.1039/C5IB00324E) 27032985

[22] Jones J, Adamatzky A 2014 Computation of the travelling salesman problem by a shrinking blob. Nat. Comput. 13, 1–16. (10.1007/s11047-013-9401-x)

[23] Coulouris GF et al. 2005 Distributed systems: concepts and design. London, UK: Pearson Education.

[24] Navlakha S, Bar-Joseph Z 2015 Distributed information processing in biological and computational systems. Commun. ACM 58, 94–102. (10.1145/2678280)

[25] Von Neumann J. 1956 Probabilistic logics and the synthesis of reliable organisms from unreliable components. Automata Stud. 34, 43–98. (10.1515/9781400882618-003)

[26] Moore EF, Shannon CE 1956 Reliable circuits using less reliable relays. J. Franklin Inst. 262, 191–208. (10.1016/0016-0032(56)90559-2)

[27] Katifori E, Szöllősi GJ, Magnasco MO 2010 Damage and fluctuations induce loops in optimal transport networks. Phys. Rev. Lett. 104, 048704 (10.1103/PhysRevLett.104.048704) 20366746

[28] Regot S et al 2011 Distributed biological computation with multicellular engineered networks. Nature 469, 207–211. (10.1038/nature09679) 21150900

[29] Brophy JA, Voigt CA 2014 Principles of genetic circuit design. Nat. Methods 11, 508–520. (10.1038/nmeth.2926) 24781324 PMC4230274

[30] Shapiro JA 1995 The significances of bacterial colony patterns. Bioessays 17, 597–607. (10.1002/bies.950170706) 7646482

[31] Czirók A, Ben-Jacob E, Cohen I, Vicsek T 1996 Formation of complex bacterial colonies via self-generated vortices. Phys. Rev. E 54, 1791 (10.1103/PhysRevE.54.1791) 9965259

[32] Prindle A, Liu J, Asally M, Ly S, Garcia-Ojalvo J, Süel GM 2015 Ion channels enable electrical communication in bacterial communities. Nature 527, 59–63. (10.1038/nature15709) 26503040 PMC4890463

[33] Couzin ID 2009 Collective cognition in animal groups. Trends Cogn. Sci. 13, 36–43. (10.1016/j.tics.2008.10.002) 19058992

[34] Bonabeau E, Marco DDRDF, Dorigo M, Théraulaz G, Theraulaz G 1999 Swarm intelligence: from natural to artificial systems. Oxford, UK: Oxford University Press.

[35] Bonabeau E, Theraulaz G, Deneubourg JL, Aron S, Camazine S 1997 Self-organization in social insects. Trends Ecol. Evol. 12, 188–193. (10.1016/S0169-5347(97)01048-3) 21238030

[36] Rivault C, Cloarec A, Sreng L 1998 Cuticular extracts inducing aggregation in the German cockroach, *Blattella germanica* (L.). J. Insect Physiol. 44, 909–918. (10.1016/S0022-1910(98)00062-6) 12770427

[37] Crall JD et al 2016 Social context modulates idiosyncrasy of behaviour in the gregarious cockroach *Blaberus discoidalis*. Anim. Behav. 111, 297–305. (10.1016/j.anbehav.2015.10.032)

[38] Bassel GW 2018 Information processing and distributed computation in plant organs. Trends Plant Sci. 23, 994–1005. (10.1016/j.tplants.2018.08.006) 30219546

[39] Smith JM 1999 Shaping life: genes, embryos and evolution. New Haven, CT: Yale University Press.

[40] Driever W, Nüsslein-Volhard C 1988 A gradient of bicoid protein in *Drosophila* embryos. Cell 54, 83–93. (10.1016/0092-8674(88)90182-1) 3383244

[41] Jernvall J, Newman SA 2003 Mechanisms of pattern formation in development and evolution. Development 130, 2027–2037. (10.1242/dev.00425) 12668618

[42] Saka Y, Lhoussaine C, Kuttler C, Ullner E, Thiel M 2011 Theoretical basis of the community effect in development. BMC Syst. Biol. 5, 54 (10.1186/1752-0509-5-54) 21496342 PMC3105943

[43] Coen E, Rolland-Lagan AG, Matthews M, Bangham JA, Prusinkiewicz P 2004 The genetics of geometry. Proc. Natl Acad. Sci. USA 101, 4728–4735. (10.1073/pnas.0306308101) 14960734 PMC387316

[44] Buckley TN 2005 The control of stomata by water balance. New Phytol. 168, 275–292. (10.1111/j.1469-8137.2005.01543.x) 16219068

[45] Peak D et al 2004 Evidence for complex, collective dynamics and emergent, distributed computation in plants. Proc. Natl Acad. Sci. USA 101, 918–922. (10.1073/pnas.0307811100) 14732685 PMC327117

[46] Topham AT et al 2017 Temperature variability is integrated by a spatially embedded decision-making center to break dormancy in *Arabidopsis* seeds. Proc. Natl Acad. Sci. USA 114, 6629–6634. (10.1073/pnas.1704745114) 28584126 PMC5488954

[47] Wolfram S 1984 Universality and complexity in cellular automata. Physica D 10, 1–35. (10.1016/0167-2789(84)90245-8)

[48] Gács P, Kurdyumov GL, Levin LA 1978 One-dimensional uniform arrays that wash out finite islands. Problemy Peredachi Informatsii 14, 92–96.

[49] Bak P, Tang C, Wiesenfeld K 1988 Self-organized criticality. Phys. Rev. A 38, 364 (10.1103/PhysRevA.38.364) 9900174

[50] Malamud BD, Morein G, Turcotte DL 1998 Forest fires: an example of self-organized critical behavior. Science 281, 1840–1842. (10.1126/science.281.5384.1840) 9743494

[51] Finch-Savage WE, Leubner-Metzger G 2006 Seed dormancy and the control of germination. New Phytol. 181, 501–523. (10.1111/j.1469-8137.2006.01787.x) 16866955

[52] Kucera B, Cohn MA, Leubner-Metzger G 2005 Plant hormone interactions during seed dormancy release and germination. Seed Sci. Res. 15, 281–307. (10.1079/SSR2005218)

[53] Bogacz R 2007 Optimal decision-making theories: linking neurobiology with behaviour. Trends Cogn. Sci. 11, 118–125. (10.1016/j.tics.2006.12.006) 17276130

[54] Bogacz R, Gurney K. 2007 The basal ganglia and cortex implement optimal decision making between alternative actions. Neural Comput. 19, 442–477. (10.1162/neco.2007.19.2.442) 17206871

[55] Thompson K. et al 1977 Seed germination in response to diurnal fluctuations of temperature. Nature 267, 147–149. (10.1038/267147a0) 16073423

[56] Levy YY, Dean C 1998 The transition to flowering. Plant Cell 10, 1973–1989. (10.1105/tpc.10.12.1973) 9836739 PMC526001

[57] Bastow R et al 2004 Vernalization requires epigenetic silencing of FLC by histone methylation. Nature 427, 164–167. (10.1038/nature02269) 14712277

[58] Antoniou-Kourounioti RL et al 2018 Temperature sensing is distributed throughout the regulatory network that controls FLC epigenetic silencing in vernalization. Cell Syst. 7, 643–655. (10.1016/j.cels.2018.10.011) 30503646 PMC6310686

[59] Angel A, Song J, Yang H, Questa JI, Dean C, Howard M 2015 Vernalizing cold is registered digitally at FLC. Proc. Natl Acad. Sci. USA 116, 4146–4151. (10.1073/pnas.1503100112) PMC4386389 25775579

[60] Wigge PA et al 2005 Integration of spatial and temporal information during floral induction in *Arabidopsis*. Science 309, 1056–1059. (10.1126/science.1114358) 16099980

[61] Moore C 1997 Majority-vote cellular automata, Ising dynamics, and P-completeness. J. Stat. Phys. 88, 795–805. (10.1023/B:JOSS.0000015172.31951.7b)

[62] Hepworth J et al 2018 Absence of warmth permits epigenetic memory of winter in *Arabidopsis*. Nat. Commun. 9, 639 (10.1038/s41467-018-03065-7) 29434233 PMC5809604

[63] Rinne PL, van der Schoot C. 1998 Symplasmic fields in the tunica of the shoot apical meristem coordinate morphogenetic events. Development 125, 1477–1485. 9502728 10.1242/dev.125.8.1477

[64] Lucas WJ, Ding B, van der Schoot C. 1993 Plasmodesmata and the supracellular nature of plants. New Phytol. 125, 435–476. (10.1111/j.1469-8137.1993.tb03897.x) 33874589

[65] Gisel A, Barella S, Hempel FD, Zambryski PC 1999 Temporal and spatial regulation of symplastic trafficking during development in *Arabidopsis thaliana* apices. Development 126, 1879–1889. 10101122 10.1242/dev.126.9.1879

[66] McLean BG, Hempel FD, Zambryski PC 1997 Plant intercellular communication via plasmodesmata. Plant Cell 9, 1043 (10.1105/tpc.9.7.1043) 9254930 PMC156978

[67] Tylewicz S et al. 2018 Photoperiodic control of seasonal growth is mediated by ABA acting on cell–cell communication. Science 360, 212–215. (10.1126/science.aan8576) 29519919

[68] Brown SD, Francis RJ, Rose J, Vranesic ZG 2012 Field-programmable gate arrays, vol. 180 Berlin, Germany: Springer Science & Business Media.

[69] Miller MB, Bassler BL 2001 Quorum sensing in bacteria. Annu. Rev. Microbiol. 55, 165–199. (10.1146/annurev.micro.55.1.165) 11544353

[70] Jackson MD, Xu H, Duran-Nebreda S, Stamm P, Bassel GW 2017 Topological analysis of multicellular complexity in the plant hypocotyl. eLife 6, e26023 (10.7554/eLife.26023) 28682235 PMC5499946

[71] Jackson MD, Duran-Nebreda S, Bassel GW 2017 Network-based approaches to quantify multicellular development. J. R. Soc. Interface 14, 20180484 (10.1098/rsif.2017.0484) PMC5665831 29021161

[72] Toyota M et al 2018 Glutamate triggers long-distance, calcium-based plant defense signaling. Science 361, 1112–1115. (10.1126/science.aat7744) 30213912

[73] Huber AE, Bauerle TL 2016 Long-distance plant signaling pathways in response to multiple stressors: the gap in knowledge. J. Exp. Bot. 67, 2063–2079. (10.1093/jxb/erw099) 26944636

[74] Nguyen CT, Kurenda A, Stolz S, Chételat A, Farmer EE 2018 Identification of cell populations necessary for leaf-to-leaf electrical signaling in a wounded plant. Proc. Natl Acad. Sci. USA 115, 10 178–10 183. (10.1073/pnas.1807049115) PMC6176584 30228123

[75] Deng Y, Liu Y, Zhou D 2015 An improved genetic algorithm with initial population strategy for symmetric TSP. Math. Probl. Eng. 2015, 212794 (10.1155/2015/212794)

[76] Ciszak M, Comparini D, Mazzolai B, Baluska F, Arecchi FT, Vicsek T, Mancuso S 2012 Swarming behavior in plant roots. PLoS ONE 7, e29759 (10.1371/journal.pone.0029759) 22272246 PMC3260168

